# Expression of the peroxisome proliferator activated receptor γ gene is repressed by DNA methylation in visceral adipose tissue of mouse models of diabetes

**DOI:** 10.1186/1741-7007-7-38

**Published:** 2009-07-10

**Authors:** Katsunori Fujiki, Fumi Kano, Kunio Shiota, Masayuki Murata

**Affiliations:** 1 Department of Life Sciences, Graduate School of Arts and Sciences, The University of Tokyo, Tokyo, Japan; 2 Department of Animal Resource Sciences, Graduate School of Agricultural and Life Sciences, The University of Tokyo, Tokyo, Japan

## Abstract

**Background:**

Adipose tissues serve not only as a store for energy in the form of lipid, but also as endocrine tissues that regulates metabolic activities of the organism by secreting various kinds of hormones. Peroxisome proliferator activated receptor γ (PPARγ) is a key regulator of adipocyte differentiation that induces the expression of adipocyte-specific genes in preadipocytes and mediates their differentiation into adipocytes. Furthermore, PPARγ has an important role to maintain the physiological function of mature adipocyte by controlling expressions of various genes properly. Therefore, any reduction in amount and activity of PPARγ is linked to the pathogenesis of metabolic syndrome.

**Results:**

In this study, we investigated the contribution of epigenetic transcriptional regulatory mechanisms, such as DNA methylation, to the expression of the PPARγ gene, and further evaluated the contribution of such epigenetic regulatory mechanisms to the pathogenesis of metabolic syndrome. In 3T3-L1 preadipocytes, the promoter of the PPARγ2 gene was hypermethylated, but was progressively demethylated upon induction of differentiation, which was accompanied by an increase of mRNA expression. Moreover, treatment of cells with 5'-aza-cytideine, an inhibitor of DNA methylation, increased expression of the PPARγ gene in a dose-dependent manner. Methylation *in vitro *of a PPARγ promoter-driven reporter construct also repressed the transcription of a downstream reporter gene. These results suggest that the expression of the PPARγ gene is inhibited by methylation of its promoter. We next compared the methylation status of the PPARγ promoters in adipocytes from wild-type (WT) mice with those from two diabetic mouse models: *+Lepr*^*db*^*/+Lepr*^*db *^and diet-induced obesity mice. Interestingly, we found increased methylation of the PPARγ promoter in visceral adipose tissues (VAT) of the mouse models of diabetes, compared to that observed in wild-type mice. We observed a concomitant decrease in the level of PPARγ mRNA in the diabetic mice compared to the WT mice.

**Conclusion:**

We conclude that the expression of PPARγ gene is regulated by DNA methylation of its promoter region and propose that reduced expression of PPARγ owing to DNA methylation in adipocytes of the VAT may contribute to the pathogenesis of metabolic syndrome.

## Background

The process of adipocyte differentiation is ascribed to the activation of the expression of adipocyte-specific genes. Several transcriptional regulators, including cytidine-cytidine-adenosine-adenosine-thymidine (CCAAT)/enhancer-binding proteins (C/EBPα, C/EBPβ and C/EBPδ) and the nuclear hormone receptor, peroxisome proliferators activated receptor γ (PPARγ), play pivotal roles in the early stages of this process [1,2]. These factors control the expression of adipocyte-specific genes by means of direct interaction with PPARγ or C/EBP binding sites within the promoters of these genes [3]. PPARγ is an important key regulator of adipogenesis. Overexpression of PPARγ induces the accumulation of lipid droplets and the generation of adipocyte-like characteristics in differentiated fibroblast cells [4]. In contrast, decreased fat mass and a smaller size of adipocytes are observed in heterozygous PPARγ-deficient mice [5]. PPARγ is crucial for adipogenesis, and PPARγ is the only factor described so far that is both necessary and sufficient to promote adipogenesis. There are two isoforms of PPARγ: PPARγ1 and PPARγ2. The latter has been shown to be an adipocyte-specific isoform and is more efficient than PPARγ1 in promoting adipocyte differentiation. PPARγ2 contains an additional 30 amino acids at its N-terminus, that is not present in PPARγ1, thus indicating that the transcription of each isoform is initiated at different start sites, and that it is differentially controlled [6,7].

Extensive studies over the past several years have revealed that adipose tissue not only has a role in storing energy as triglycerides, but also has an important role in controlling overall metabolism through the secretion of various hormones called adipokines [8,9]. The secretion profile of these adipokines is altered by obesity, which is characterized by both hypertrophy and hyperplasia of adipocytes. This change in adipokine secretion first leads to insulin resistance, which represents an important component of the development of metabolic syndrome as typified by type 2 diabetes mellitus [10,11]. Obese conditions induce a decline in the activity and amount of PPARγ, the master regulator of adipogenesis, which disturbs adipocyte metabolism. Various changes in the activity and expression of PPARγ in obesity have been reported, including a decrease in transcription and translation [12-14], activation of the degradation of both its mRNA and protein [11], and a decline of the ligand-binding affinity of PPARγ by post-translational modification [15]. Although the details of the regulatory mechanisms controlling the activity and the amount of PPARγ at the various levels remains unclear, these alterations appear to be strongly associated with the pathogenesis of metabolic syndrome. To date, thiazolidinediones (TZDs), PPARγ agonists, are some of the more effective treatments used for improvement of insulin resistance.

DNA methylation results from the transfer by DNA methyltransferase of a methyl group from S-adenosylmethionine to the cytosine residue within CpG dinucleotides. DNA methylation has been established to be an important epigenetic marker of the transcriptionally repressed state of the genes [16,17]. There are two general mechanisms by which DNA methylation inhibits gene expression. The first is that modification of cytosine bases can directly inhibit the association of DNA binding factors owing to steric hindrance, and the second, more important mechanism is where various proteins that recognize methylated CpG sites recruit transcriptional corepressor molecules so as to silence transcription. Those corepressors, such as histone modification enzymes and chromatin remodeling enzymes, can function independently or may act in concert with factors mediating DNA methylation so as to generate the transcriptionally repressed structure of chromatin [18]. Several studies have reported that epigenetic regulatory mechanisms are involved in the transcriptional activation of PPARγ2 during adipogenesis [19]. During the differentiation of 3T3-L1 preadipocytes to adipocytes, the SWI/SNF (for 'SWItch/Sucrose NonFermentable') chromatin remodeling complex and histone modifying enzymes lead to chromatin remodeling that is followed by the sequential binding of *Pol*II, TATA binding protein (TBP) and other transcription factors to activate the transcription of PPARγ [20]. However, few studies have clearly demonstrated an active contribution of DNA methylation of the PPARγ promoter to its expression in adipocytes [21,22].

In this report, we demonstrate that the expression of the PPARγ2 gene is affected by DNA methylation of its promoter. We show that transcription of PPARγ was repressed in 3T3-L1 preadipocytes and that its promoter was methylated. During adipogenesis, activation of PPARγ expression was associated with demethylation of its promoter. The activation of the endogenous gene in cultured cells by treatment with a DNA methylation inhibitor and the reduced luciferase reporter gene expression from an *in vitro *methylated PPARγ reporter plasmid provided supporting evidence that the expression of PPARγ mRNA is controlled by DNA methylation of its promoter. We also demonstrate that methylation of the PPARγ2 promoter was increased in visceral adipose tissues of obese diabetic mouse models compared to that observed in wild-type mice. There was a concomitant reduction in PPARγ mRNA in the obese mice compared to the level in wild-type mice. Taken together, we propose that perturbation of epigenetic regulation of the PPARγ2 gene during obesity causes a reduction in the expression of PPARγ, which might contribute to the pathogenesis of metabolic syndrome.

## Results

### DNA demethylation of PPARγ promoter in 3T3-L1 adipocyte differentiation

**Figure 1:**
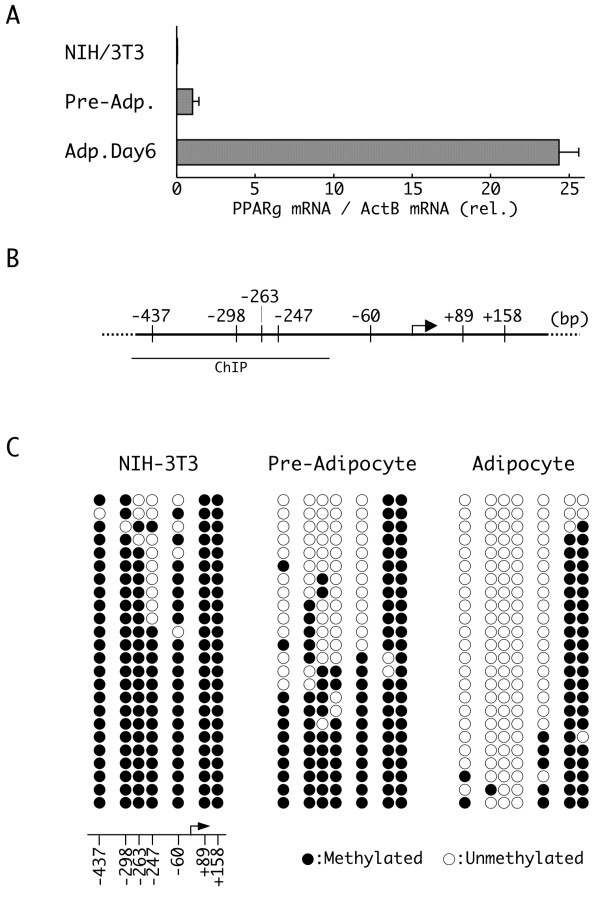
**Expression of peroxisome proliferators activated receptor γ (PPARγ) mRNA and differential methylation of the PPARγ promoter**. **(a) **Relative expression of PPARγ mRNA in NIH/3T3, 3T3-L1 preadipocytes (day 0) and differentiated 3T3-L1 adipocytes (day 6). The expression of PPARγ and β-actin mRNAs were determined by real time reverse transcriptase polymerase chain reaction (RT-PCR). The level of PPARγ mRNA was normalized to that of β-actin, and the relative normalized levels are shown (n = 3, mean ± SD). **(b) **A schematic diagram of the PPARγ promoter. An arrow indicates the transcription start site (TSS) (+1 bp), and short vertical lines indicate the positions of the methylation sites relative to the TSS. The region detected by the chromatin immunoprecipitation (ChIP) analysis is indicated below the schematic (see Figure 4). **(c) **Bisulfite sequencing analysis of the DNA methylation profile of the individual CpG sites in the PPARγ promoter in NIH/3T3, 3T3-L1 preadipocytes (day 0) and 3T3-L1 adipocytes (day 6). Each PCR product was subcloned, and eight clones were subjected to sequencing analysis. The data represent the aggregate total of three independent experiments. The methylation status of each site, either methylated (closed circle) or unmethylated (open circle), is aligned corresponding to their genomic order (represented at the bottom of the results for NIH/3T3 cells).

Since PPARγ is the key regulator of adipogenic differentiation, its transcription is highly restricted in most of cell types [4]. Real time reverse-transcription polymerase chain reaction (RT-PCR) of total PPARγ mRNA revealed that its expression is strongly inhibited in NIH/3T3 fibroblasts and somewhat less so in 3T3-L1 preadipocytes (Figure 1a). Expression of PPARγ mRNA in NIH/3T3 cells was approximately 3% of that observed in 3T3-L1 preadipocytes. In contrast, expression of PPARγ mRNA in differentiated 3T3-L1 adipocytes (day 6) was approximately 24 times greater than that observed in 3T3-L1 preadipocytes. To examine the contribution of epigenetic factors, such as DNA methylation, on the transcriptional regulation of PPARγ expression, we first compared the methylation status of the PPARγ promoter region in NIH-3T3 cells, preadipocytes (day 0) and adipocytes (day 6) using the bisulfite sequencing method. There are seven CpG methylation sites flanking the transcription start site (TSS) of the murine PPARγ2 gene (Figure 1b). We examined the methylation status of these seven sites in promoter fragments isolated from 24 cells of each cell type (Figure 1c). In NIH/3T3 cells, which express a low level of PPARγ mRNA, almost all of these sites were methylated. A significant fraction of these sites were also methylated in 3T3-L1 preadipocytes, although the extent of methylation was lower than that observed in NIH/3T3 cells. In contrast, the CpG sites located upstream of the TSS in 3T3-L1 adipocytes were mostly demethylated after differentiation to adipocytes. These results indicated that methylation of the CpG sites upstream of the TSS correlates inversely with PPARγ mRNA expression. Thus, CpG methylation of these sites in the PPARγ promoter might contribute to silencing of its expression. The two CpG sites located downstream of the TSS, at positions +89 and +158, were methylated in each cell type suggesting that methylation of these CpG sites does not contribute to the regulation of PPARγ mRNA expression.

### Contribution of DNA methylation to PPARγ expression

**Figure 2:**
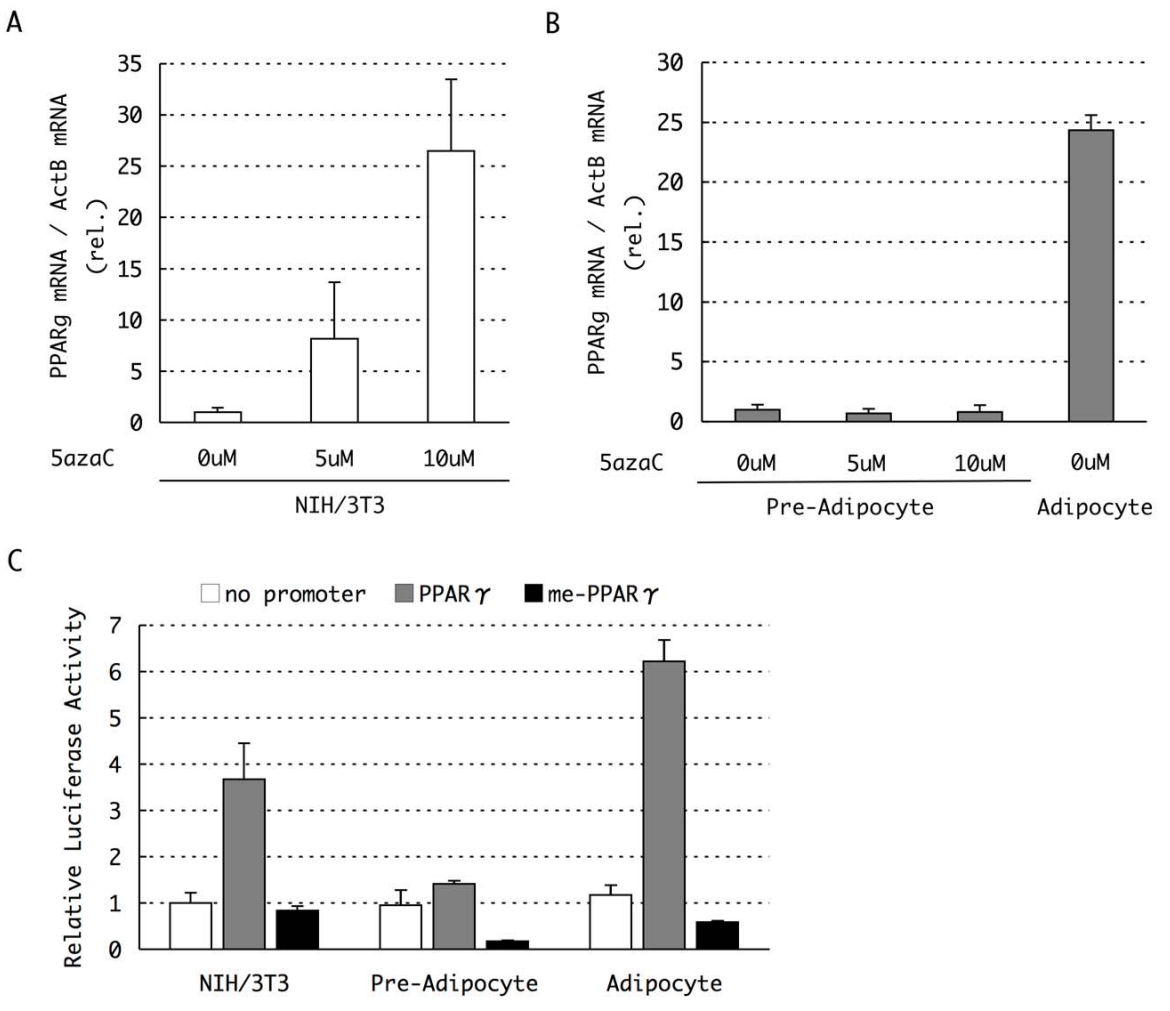
**Activation of peroxisome proliferators activated receptor γ (PPARγ) gene by 5'-aza-cytideine (5'-aza-C) and evaluation of promoter activity with a luciferase assay**. **(a, b) **Expression of PPARγ mRNA in 5'-aza-C-treated NIH/3T3 and 3T3-L1 preadipocytes. The cells were cultured in growth medium containing 5'-aza-C at the indicated concentrations for 48 h before harvest. The mRNA expression levels were determined by real time reverse transcriptase polymerase chain reaction (RT-PCR) and normalized to the levels of β-actin mRNA measured in parallel experiments (n = 3, mean ± SD). The expression level of PPARγ mRNA in differentiated day 6 3T3-L1 adipocytes was also presented at the right of **(b) **for comparison. **(c) **Luciferase expression from PPARγ promoter reporter constructs in the presence or absence of *in vitro *DNA methylation in NIH/3T3, 3T3-L1 preadipocytes and differentiating adipocytes (day 4). Approximately 1 kb of the region upstream of the PPARγ transcription start site (TSS) was cloned into a luciferase reporter vector, and the vector was methylated *in vitro *as needed. Relative luciferase activity, normalized to the activity of a cotransfected internal control vector, is shown (n = 3, mean ± SD).

To further evaluate a causal relationship between methylation of the PPARγ promoter and the expression of PPARγ mRNA, we examined the effect of 5'-aza-cytideine (5'-aza-C), an inhibitor of DNA methylation, on PPARγ mRNA expression in NIH/3T3 cells. After 48 h treatment with 5 and 10 μM 5'-aza-C, PPARγ expression was analyzed quantitatively by real time RT-PCR. As shown in Figure 2a, the expression of PPARγ mRNA increased following 5'-aza-C treatment in a dose-dependent manner. In contrast, treatment of cells with trichostatin A (TSA), an inhibitor of histone deacetylases, for 48 h, had no effect on PPARγ mRNA expression (Additional file 1). These results suggest that DNA methylation is an important mechanism of epigenetic regulation of the expression of PPARγ and that the role of DNA methylation is dominant to that of histone acetylation, at least under these experimental conditions tested here.

To further confirm the effect of DNA methylation on the PPARγ promoter region, we performed luciferase reporter assays in NIH/3T3 cells following transfection of a reporter plasmid comprising 1 kb of the 5'-upstream region of PPARγ containing five CpG sites (at positions -437 to -60, Figure 1b) placed upstream of a luciferase reporter gene. Transfection of the PPARγ reporter construct led to the expression of 3.7-fold more luciferase activity relative to that of the empty vector (Lacking a promoter upstream of the reporter gene) (Figure 2c). In contrast, *in vitro *DNA methylation of the reporter construct prior to transfection reduced the expression of the luciferase reporter to a level similar to that observed when using the empty vector (Figure 2c). These results further suggest that transcription of the PPARγ gene is regulated through CpG methylation of the promoter.

The same 5'-aza-C treatment and luciferase assays were next performed on 3T3-L1 preadipocytes (day 0) and adipocytes (day 4). In contrast to the results observed in NIH/3T3 cells, we observed no increase in PPARγ mRNA in 5'-aza-C-treated preadipocytes (Figure 2b). This result suggested that, in preadipocytes, DNA demethylation alone is not sufficient for the activation of the PPARγ promoter. Similar results were obtained from following luciferase reporter assays in preadipocytes (Figure 2c). The unmethylated PPARγ reporter construct did not give rise to increased luciferase expression and instead expressed a similar level of luciferase as the promoterless construct in preadipocytes. Luciferase expression from the promoterless construct in preadipocytes was similar to the level observed in NIH/3T3 cells. These results suggested the existence of an inhibitory mechanism that represses expression from the demethylated PPARγ promoter in preadipocytes, or alternatively, an activating mechanism that induces the expression of the demethylated PPARγ promoter in NIH/3T3 cells.

Luciferase expression from the *in vitro *methylated reporter construct was strongly repressed in preadipocytes (Figure 2c). Indeed, luciferase reporter expression from the methylated PPARγ construct was further reduced to 18% of that of the promoterless control construct, suggesting that preadipocytes inhibit transcription from the methylated PPARγ promoter more robustly than NIH/3T3 cells. These data further support the idea that the expression from the PPARγ promoter is under the control of its promoter methylation.

As was expected, luciferase reporter expression from the unmethylated PPARγ reporter vector was very efficient in differentiating adipocytes (day 4), in which the endogenous PPARγ gene is activated (Figure 2c). In contrast, luciferase reporter expression from the *in vitro *methylated promoter construct remained low in adipocytes, despite the presence of a suitable environment for activation of the transcription of the PPARγ promoter. These results further suggest that DNA methylation contributes to the regulation of expression of PPARγ mRNA. In addition, although there are structural differences between endogenous chromatin and exogenous reporter plasmids, DNA methylation of the PPARγ promoter may be a dominant regulatory mechanism that can override activation of the PPARγ promoter by other transcription factors during adipogenesis.

### Kinetic analysis of PPARγ promoter demethylation

**Figure 3:**
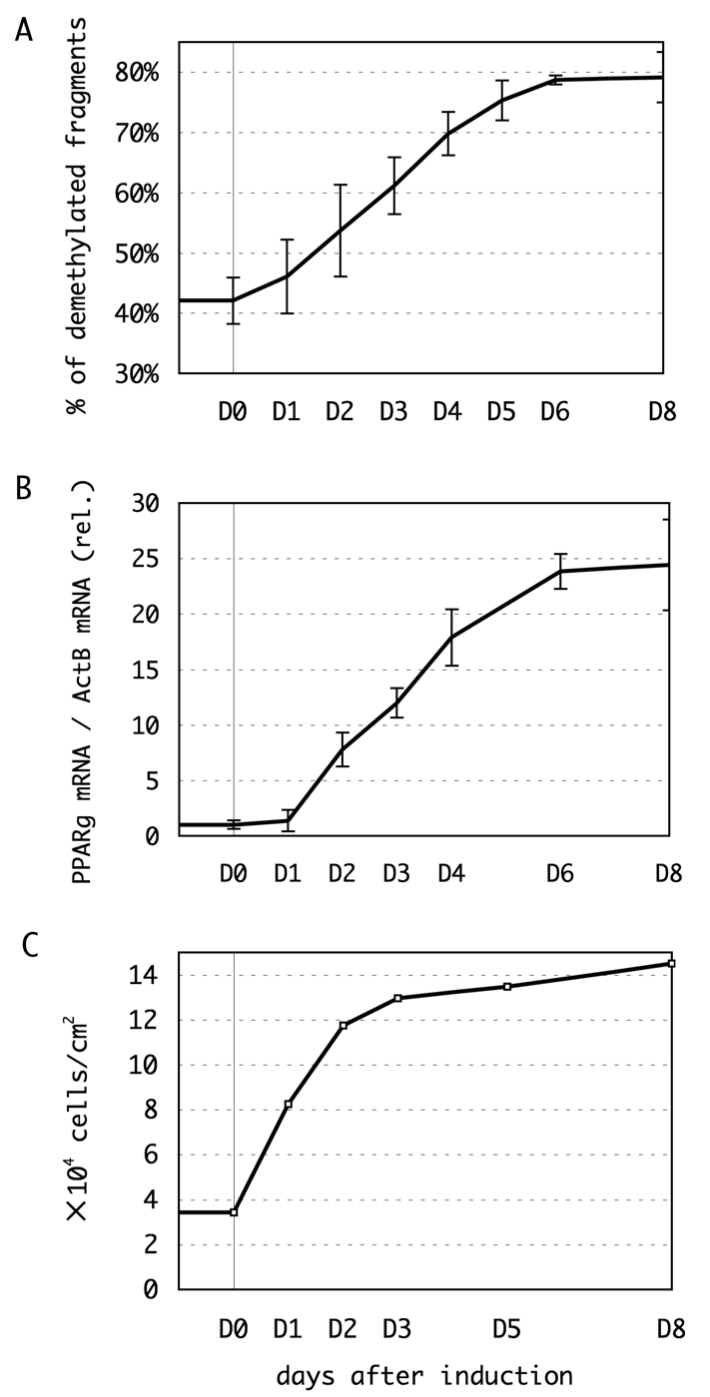
**Kinetic analysis of peroxisome proliferators activated receptor γ (PPARγ) promoter demethylation, mRNA expression and cell proliferation**. **(a, b) **The timelapse analysis of PPARγ promoter demethylation and mRNA expression during adipogenesis. Preadipocytes were stimulated to differentiate on day 0 (D0), and harvested every 24 or 48 h until day 8 (D8). The methylation status of the -437 bp, -298 bp and -247 bp CpG sites were determined by restriction endonuclease digestion, and the fraction of the promoter fragments in which all of the three sites were unmethylated is represented **(a)**. The mRNA expression levels were measured by real time reverse transcriptase polymerase chain reaction (RT-PCR) and normalized to the level of β-actin mRNA **(b)**. Individual assessments were repeated three times and the means ± SD are represented, respectively. **(c) **The increase in cell number by mitotic clonal expansion. The number of differentiating cells was counted at the indicated timepoints, and the cell density was calculated.

For further analysis of PPARγ methylation in adipocytes, we next investigated the time course of promoter demethylation during adipocyte differentiation of 3T3-L1 cells. Bisulfite-converted PCR amplicons of the PPARγ promoter of the cells were digested with the restriction enzyme, *Hpy*CH4IV. The level of demethylation was estimated by the cutting efficiency every 24 h (see Methods for details). We could detect demethylation of the promoter immediately following induction of differentiation and the demethylation increased gradually until it reached a plateau on day 6 (Figure 3a).

The level of PPARγ mRNA was also monitored by real time RT-PCR (Figure 3b). Similar to the time course of demethylation of the promoter, the amount of PPARγ mRNA gradually increased until it reached a plateau on day 6, although the starting time of the increase in mRNA appeared to lag 1 to 2 days behind the start of promoter demethylation and differentiation induction. This synchronization supports the idea that transcriptional activation of the PPARγ gene is regulated by DNA methylation/demethylation. The observed lag time probably indicates that promoter demethylation is not the only factor controlling PPARγ expression. The differentiation stimulus induces the recruitment of several transcriptional regulators that increase the expression of PPARγ, including C/EBPs, sterol regulatory element binding protein (SREBP), and SWI/SNF family chromatin remodeling enzymes to the PPARγ promoter, and the observed lag in PPARγ expression might be due to a requirement to recruit these factors to the promoter so as to activate transcription following DNA demethylation [1,19,20].

During adipogenesis of 3T3-L1 cells, genomic DNA is newly synthesized during two cycles of mitosis termed the mitotic clonal expansion (MCE) [23]. The replication of methylated DNA produces hemimethylated CpG sites, which could cause loss of DNA methylation if those sites are not remethylated by Dnmt1, the maintenance DNA methyltransferase [17]. We next investigated whether the demethylation of the PPARγ promoter during differentiation is caused by passive demethylation during DNA replication through MCE. To do this, we compared cell growth and demethylation of the PPARγ promoter during differentiation (Figure 3c). The number of the cells increased immediately following induction of differentiation, expanding fourfold by day 2, suggesting that two cycles of cell division had occurred. In contrast, as shown in Figure 3a, DNA demethylation increased gradually up day 6, and we observed no immediate demethylation that corresponding to the increase in cell number on approximately day 2. This result demonstrates that PPARγ promoter demethylation is not a passive process caused by MCE, but that it is instead an active process of epigenetic regulation of the transcriptional activity of the PPARγ promoter.

### Chromatin immunoprecipitation assays of the PPARγ promoter during 3T3-L1 adipocyte differentiation

**Figure 4:**
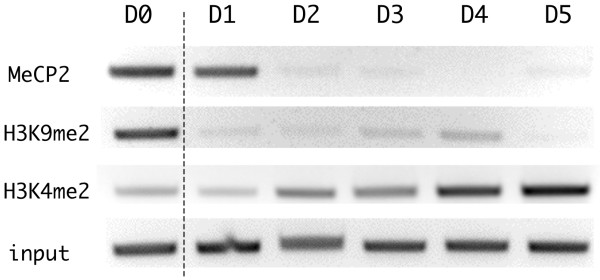
**Chromatin immunoprecipitation (ChIP) assays of the peroxisome proliferators activated receptor γ (PPARγ) promoter region during adipogenesis**. 3T3-L1 cells were harvested at the indicated times, and 106 cells were used as the input for each assay. DNA fragments immunoprecipitated by the indicated antibody were recovered and amplified by the primers designed for the PPARγ promoter region (see Figure 1). A total of 1% of the input was also amplified without ChIP, and is shown at the bottom.

Promoter DNA methylation represses the transcription of a downstream gene both directly and indirectly. Methylated CpG sites within the promoter itself can directly inhibit the binding of transcription factors due to steric hindrance and thereby repress transcription. Alternatively, methylated CpG binding domain (MBD) proteins, which specifically bind to methylated CpG residues, recruit other enzymes that modify histone tails or chromatin structure, so as to indirectly create a repressed state of chromatin [16-18]. To assess the protein interactions on the methylated PPARγ promoter region, we performed chromatin immunoprecipitation (ChIP) assays to evaluate the binding of certain MBD proteins to the PPARγ promoter. The ChIP assay revealed that methyl CpG binding protein 2 (MeCP2) was associated with the methylated PPARγ promoter in preadipocytes (day 0) (Figure 4). We were unable to detect the binding of other MBDs, such as MBD1 and MBD2a, to the promoter in preadipocytes (data not shown). In contrast, we detected the binding of MBD2a to the promoter in NIH/3T3 cells, suggesting that MBD binding to the PPARγ promoter differs between NIH/3T3 and 3T3-L1 cells (Additional file 2).

Dissociation of MeCP2 from the promoter was nearly complete within 1 to 2 days following the induction of differentiation (Figure 4), although promoter demethylation continued gradually until it reached a plateau on day 6 (Figure 3a). An increase in the level of histone H3 acetylation in the PPARγ promoter region has been reported until day 2 [20], and MeCP2 has been shown to recruit the protein complex including histone deacetylases to methylated CpG sites [18]. Thus, the increase in histone acetylation is likely caused by dissociation of MeCP2 on days 1 to 2 following the induction of differentiation. This result further suggests that DNA methylation plays a greater role than histone acetylation on the regulation of the expression of PPARγ mRNA.

We next evaluated other types of histone modifications in 3T3-L1 cells following the induction of differentiation. On day 0, we observed dimethylation of histone H3 on lysine 9 (H3K9me2), which represents a repressed state of chromatin, on the PPARγ promoter. This dimethylation disappeared within 1 day of the induction of differentiation (Figure 4). The demethylation of H3K9me2 occurred at a stage in the differentiation process that preceded DNA demethylation, the expression of PPARγ and even MCE (Figure 3), and so therefore was not synchronous with PPARγ expression and therefore was not responsible for the induction of its expression. This result suggests that demethylation of H3K9me2 is insufficient to induce the expression of PPARγ mRNA. In contrast, the level of dimethylation of lysine 4 on histone H3 (H3K4me2), which represents an activated state of chromatin, was low prior to the induction of differentiation, but increased gradually following induction (Figure 4). The kinetics of this gradual increase appeared to mirror that of the DNA demethylation and the increase in mRNA expression, suggesting that H3K4 dimethylation correlates well with DNA demethylation.

### DNA methylation and mRNA expression of PPARγ in white adipose tissues of mouse models of diabetes

PPARγ not only acts to induce adipogenesis, but also to maintain the functional phenotype of adipocytes. Thus, the expression and epigenetic regulation of PPARγ mRNA might also be important for the maintenance of the adipocyte phenotype, and any defect in this regulation could become a pathogenic factor in metabolic syndromes. In other words, changes in the epigenetic status of the PPARγ gene might be observed in adipose tissues under pathogenic conditions. To test this possibility, we analyzed methylation of the PPARγ promoter in white adipose tissues (WAT) and compared the methylation profiles of the promoter in wild-type mice and mouse models of diabetes. WAT are classified into two main types: subcutaneous adipose tissue (SAT) and visceral adipose tissue (VAT). Visceral obesity has been linked strongly to diabetic insulin resistance [24]. Therefore, we compared the methylation status of the PPARγ promoter in both types of WAT.

**Figure 5:**
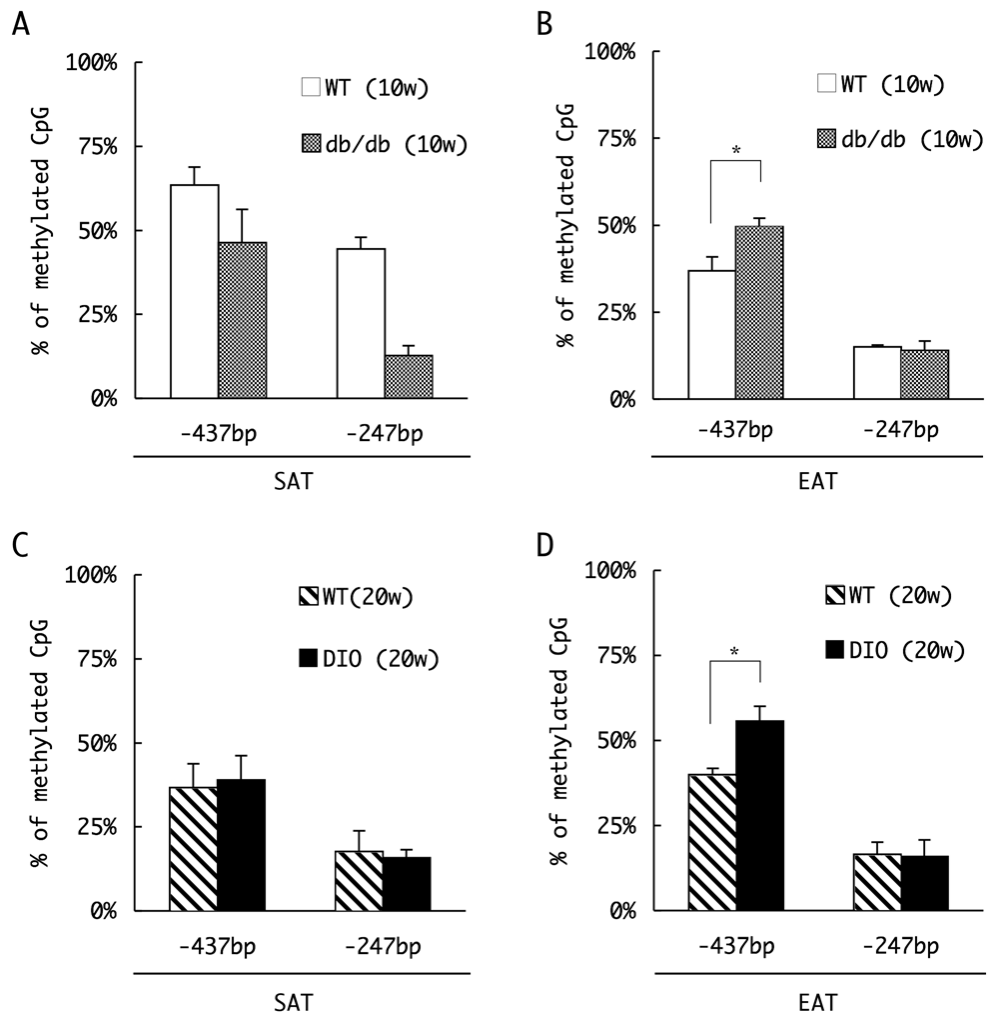
**Comparison of the DNA methylation profile of the peroxisome proliferators activated receptor γ (PPARγ) in white adipose tissue (WAT)**. Genomic DNA was extracted from subcutaneous adipose tissue (SAT) **(a, c) **and epididymal adipose tissues (EAT) **(b, d) **of 10 week-old wild-type (WT) or db/db mice **(a, b) **or 20 week-old WT/diet-induced obesity mice **(c, d)**. Genomic DNA prepared from each tissue was treated with sodium bisulfite, and amplified by polymerase chain reaction (PCR) with the primers designed for the flanking regions of the -437 bp or -247 bp CpG site. The methylation status of each site was estimated by the efficiency of restriction endonuclease digestion of the PCR amplicon, and the percentages of the methylated fragments are represented (n = 3, mean ± SD, *P < 0.05, t test).

We first compared promoter methylation in WAT isolated from 10-week-old wild-type (WT) and *+Lepr*^*db*^*/+Lepr*^*db *^(db/db) mice. The db/db mouse is a well established model of diabetes that contains a homozygous mutation in the leptin receptor gene. This mouse exhibits a phenotype similar to type 2 diabetes mellitus owing to hyperphagia and disrupted metabolism of adipocytes. We prepared genomic DNA from the two types of WAT: the inguinal part of the SAT and the visceral, epididymal adipose tissues (EAT). The genomic DNA was treated with sodium bisulfite, and the methylation status of two CpG sites within the PPARγ promoter, at positions -437 bp and -247, was estimated based on the efficiency of *Hpy*CH4IV restriction enzyme digestion (see Methods for detail) (Figure 5a, b). As shown in Figure 5a, the fraction of methylated CpG sites at both positions -437 bp and -247 in SAT was reduced in db/db mice compared to WT mice. The -247 site in particular was methylated to a much lesser extent in db/db mice (12.7%) than in WT mice (approximately 50%) (Figure 5a). Since the SAT in db/db mice was greatly enlarged relative to that in WT mice because of hyperphagia (approximately × 6.7 by weight, Additional file 3), the reduced level of methylation of the CpG sites in the PPARγ promoter in the SAT of db/db mice relative to the level in WT mice suggests a possible enrichment of differentiated, PPARγ-expressing adipocytes in the SAT of db/db mice.

**Figure 6:**
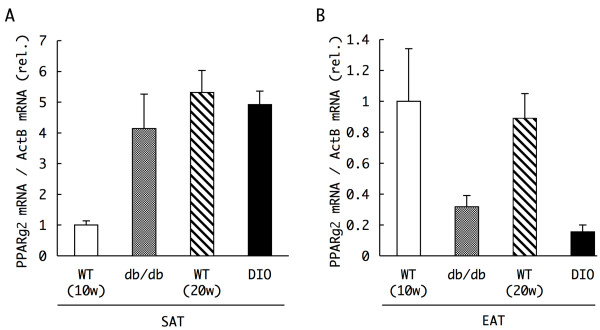
**Expression levels of peroxisome proliferators activated receptor γ (PPARγ) mRNA in white adipose tissues of normal and diabetic mice**. Total RNA was extracted from subcutaneous adipose tissue (SAT) **(a) **and epididymal adipose tissues (EAT) **(b) **of 10 week-old wild-type (WT) or db/db mice, and 20 week-old WT or diet-induced obesity mice. The mRNA expression levels of PPARγ2 were determined by real time reverse transcriptase polymerase chain reaction (RT-PCR), and normalized to that of β-actin measured in parallel as internal controls. Data represent the relative values of the mean ± SD of three independent experiments performed in triplicate.

Interestingly, despite the hypertrophy of the EAT in db/db mice (× 6.0 compared to WT), methylation of the CpG at position -437 in the PPARγ promoter was increased somewhat in db/db mice relative to the level observed in WT mice (Figure 5b). Approximately 36% of the cytosines at the site were methylated in WT mice, whereas 50% of such sites were methylated in db/db mice. The level of expression of PPARγ2 mRNA in the SAT and EAT differed between WT and db/db mice to an extent that mirrored qualitatively the differences in methylation (Figure 6a, b, WT and db/db). Whereas the expression of PPARγ2 mRNA in the SAT of db/db mice was four times greater than that in WT mice, the level of PPARγ mRNA in the EAT of db/db mice was about one-third of that observed in WT mice, which correlated inversely with the level of methylation within the PPARγ promoter, particularly with that at the CpG at -437 bp.

To our surprise, the hypertrophic EAT in db/db mice, which likely contains a large number of differentiated adipocytes, expressed a decreased level of PPARγ mRNA compared to that observed in the smaller EAT in WT mice, presumably due to the greater extent of methylation of the PPARγ promoter in the EAT of db/db mice. A reduction in the level and/or activity of PPARγ has been previously suggested to be linked to the development of diabetic symptoms such as insulin resistance [10,11]. These results suggest that enhanced methylation of the PPARγ promoter and the concomitant reduction of PPARγ mRNA in the EAT may be linked causally to the diabetic phenotype induced by obesity in db/db mice.

To further characterize this correlation, we next analyzed methylation of the PPARγ promoter in adipocytes from a mouse model of diet-induced obesity (DIO), which are WT mice fed with a high-fat diet from 4 to 20 weeks old that subsequently present a type 2 diabetes-like phenotype. Although DIO mice also had an excessive amount of SAT owing to their diet (approximately × 7.6 in weight compared to the SAT of WT mice, Additional file 3), the level of methylation of the PPARγ promoter in the SAT of these mice was similar to that observed in the SAT of 20-week-old WT mice (Figure 5c). The level of expression of PPARγ2 mRNA was similar in the SAT of DIO and WT mice (Figure 6a).

However, cells of the hypertrophic EAT of DIO mice (approximately × 3.1 compared to the EAT of WT mice) also exhibited enhanced methylation of the CpG at position -437 than was observed in cells of the smaller EAT of WT mice (Figure 5d), as had been observed in cells of the EAT of db/db mice relative to that observed in WT mice (Figure 5b). Approximately 40% of the cytosines at the site were methylated in WT mice, whereas 56% of such sites were methylated in DIO mice. Consistent with these findings, the expression of PPARγ2 mRNA was similarly reduced in the EAT of DIO mice (Figure 6b). These results suggest that, as in the EAT of db/db mice, PPARγ mRNA expression is reduced owing to promoter DNA methylation in the enlarged EAT of mice fed the high-fat diet, and further support the possibility that increased methylation of the PPARγ promoter in the obese EAT contributes to the pathogenesis of diabetes.

## Discussion and conclusion

In the present study, we found that the promoter of the PPARγ2 gene is demethylated during adipogenesis of 3T3-L1 cells, and confirmed that the expression of the PPARγ2 gene is under the control of DNA methylation within its promoter region. Although a previous study reported that the PPARγ2 promoter is differentially methylated in a tissue-dependent manner [22], a correlation between the promoter methylation and the expression has not previously been established. Here, we showed that the methylated promoters of 3T3-L1 preadipocytes were site-specifically demethylated following the induction of differentiation, and that the expression of PPARγ mRNA increased as the demethylation proceeded. The demethylation of the endogenous gene observed in cultured cells treated with 5'-aza-cytidine and the inhibition of the expression of a luciferase reporter gene following *in vitro *methylation of the PPARγ reporter plasmid prior to transfection provided supporting evidence that the expression of PPARγ mRNA is controlled by DNA methylation of the PPARγ promoter.

Our luciferase reporter assays revealed that DNA methylation of the PPARγ promoter could repress transcription of a downstream reporter gene even in differentiating adipocytes (Figure 2c, adipocyte). This finding indicates the dominant inhibitory effect of DNA methylation over the action of transcription factors that bind the promoter sequence to induce the expression of PPARγ. Since the reporter plasmids do not exhibit a higher order chromatin structure similar to genomic DNA, the dominance of DNA methylation over the chromatin-related factors that induce mRNA expression, such as histone modifications and chromatin remodeling enzymes, remains to be demonstrated. Although controversial, the results of the ChIP assays (Figure 4) and the results following TSA treatment of the cells (Additional file 1) provided additional evidence that promoter methylation works predominantly to some of these factors such as histone acetylation and dimethylation of H3K9.

We have not yet tested whether the expression of the human PPARγ gene is under the control of promoter methylation. Previous studies reported that CpG sites upstream of the TSS of the human PPARγ2 gene are differentially methylated in different cell types. The CpG sites were hypomethylated in stromal vascular cells isolated from human adipose tissue and hypermethylated in T cells that do not express PPARγ2 [21,22,25]. However, the study also reported no significant alteration in the methylation of the PPARγ promoter following adipogenesis of the stromal cells [21]. Sequence alignment of the human and murine PPARγ2 promoters reveals that most of the methylation sites are either conserved or located within approximately 25 bp upstream or downstream of each other (Additional file 4). Taken together, we hypothesize that the human PPARγ2 gene is also regulated by DNA methylation, although the human promoter contains additional CpG sites approximately 500 bp upstream of the TSS that do not exist in the murine gene.

In this study, we first investigated and compared the DNA methylation of the PPARγ gene in SAT and VAT. In the case of SAT, the age (in weeks) of the mice and the amount of differentiated cells in the tissue seemed to affect the methylation profile of PPARγ promoter (Figure 5a, c). The level of promoter methylation observed in cells of the smaller SAT from 10 week-old WT mice was greater than that observed in the larger, more differentiated SAT of db/db mice or in the SAT of 20 week-old WT and DIO mice.

In contrast, the extent of PPARγ promoter methylation observed in adipocytes of the EAT seemed to be controlled predominantly by the animals' dietary habits, such as the hyperphagia in *+Lepr*^*db*^*/+Lepr*^*db *^mice and the high-fat diet in diet-induced obesity mice (Figure 5b, d). These dietary habits increased methylation of the PPARγ promoter at position -437 in these diabetic mouse models. Corresponding decreases were observed in the expression of PPARγ2 mRNA (Figure 6b). The increase in methylation of the PPARγ promoter was unexpected, because we anticipated that the hypertrophic EAT of the diabetic mice would contain a greater number of differentiated adipocytes, and thus present a more highly demethylated promoter. Although we have not yet examined the methylation status of other CpG sites, such as those at positions -298, -263, -60, +89 and +158, the level of methylation at these sites may be similar or increased in the db/db and DIO mice relative to the levels in WT mice, since we detected reduced PPARγ mRNA expression in these diabetic mouse models.

The mechanism behind the difference in the methylation status of the PPARγ promoter in cells of the EAT between WT and diabetic mice remains unclear. The obesity-inducing dietary habits of the model mice causes various changes such as endoplasmic reticulum (ER) stress and oxidative stress caused by hypertrophy of adipocytes, an alteration of the composition of accumulated fat, or increase of free fatty acid in blood [11,26,27]. Some of these factors might have affected the observed methylation excess in the EAT of the diabetic mice.

Three possible hypotheses about the mechanism can be considered. The first hypothesis is that the population of undifferentiated preadipocytes, whose PPARγ promoters are methylated, increases in the EAT leading to an excess of promoter methylation in the whole tissue. The increase in the population of undifferentiated preadipocytes might represent an adaptation of the tissue to the high-load dietary habits, through the generation of increased numbers of preadipocytes that could subsequently differentiate into adipocytes that are capable of storing triglycerides. Otherwise, obesity-inducing dietary habits might lead to the accumulation of preadipocytes in the EAT whose potential for differentiation is impaired, such that the EAT might accumulate preadipocytes in which the PPARγ promoter is methylated, leading to an excess of methylation in the whole tissue. Adipocytes secrete a large amount of the inflammatory cytokine, tumor necrosis factor α (TNFα) during the development of obesity, which contributes to the induction of insulin resistance [8]. TNFα can also prevent the differentiation of cultured 3T3-L1 cells and human primary adipocyte precursor cells [28,29]. Thus, TNFα secreted in obese animals might inhibit the differentiation of preadipocytes in the EAT and result in the accumulation of preadipocytes.

The second hypothesis is that remethylation or impaired demethylation of the PPARγ promoter may occur in adipocytes of the EAT of diabetic mice. The remethylation of the demethylated promoter in differentiated adipocytes might represent an adaptation of obese adipocytes that are no longer able to accumulate lipid by reducing the expression of PPARγ, which would otherwise continue to promote lipid synthesis and accumulation. The third hypothesis is that the obese condition induces an increase in cell populations other than adipocytes in the EAT of diabetic mice. For example, obese EAT may require a greater number of vascular cells for supplying blood to the developing tissue. Obesity also induces macrophage infiltration of the stroma of adipose tissue [30,31]. At this time, we have no data regarding the cell types that specifically accumulate in obese EAT. However, we exclusively detected the changes of PPARγ2 mRNA in the presented experiments, and no significant difference was observed in the expression of PPARγ1, the PPARγ that the other cell types, such as vascular cells and macrophages, mainly express (data not shown). Additional histological and immunohistochemical analyses of tissue sections that evaluate the precise cellular populations of preadipocytes, adipocytes and others in the obese tissue should provide additional information that may help determine which of these three hypotheses is more likely to explain the unexpected excess in the level of PPARγ promoter methylation in the EAT of diabetic mice.

Obesity-induced alterations in the metabolism of adipose tissue, such as reprogramming of the adipokine secretion profile, is known to contribute to the pathology of insulin resistance leading to metabolic syndrome. Thiazolidine derivatives are PPARγ agonists that are used to treat patients with type 2 diabetes mellitus. These agents improve the perturbed adipokine secretion and the insulin resistance, thereby further implicating a decline in the activity and level of PPARγ in the pathogenesis of diabetes [10,11]. In this study, we have shown that alteration of the adipokine secretion profile as a result of obesity might be attributable, at least partially, to the decreased expression of PPARγ mediated by he epigenetic changes in the VAT. Although the mechanism behind the excess of DNA methylation remains to be demonstrated, this finding and following researches into the mechanism will provide a new knowledge of the pathogenesis of metabolic syndrome.

## Methods

### Cell culture, differentiation conditions and tissue preparation

All cell types were maintained at 37°C in humidified air with 5% CO_2_. 3T3-L1 preadipocytes were cultured in Dulbecco's modified Eagle medium (DMEM, Nissui, Taito-ku, Tokyo, Japan) supplemented with 10% calf serum (CS, Gibco Invitrogen, Carlsbad, California, USA) (growth medium). Growth medium was replaced every 2 days. Cells were cultured for an additional day after reaching confluency, and the growth medium was then replaced (day 0) with DMEM supplemented with 10% fetal calf serum (FCS, Gibco Invitrogen, Carlsbad, California, USA), 10 μg/ml insulin (Sigma, St. Louis, Missouri, USA), 0.5 mM 3-isobutyl-1-methylxanthine (Sigma), and 0.1 μM dexamethasone (Sigma) (differentiation medium). After 48 h, the differentiation medium was replaced (day 2) with DMEM + 10% FCS containing 5 μg/ml insulin, and the cells were allowed to accumulate lipid droplets until experimental use. NIH/3T3 cells were cultured in DMEM + 10% FCS.

To measure effects of DNA methylation and histone acetylation on PPARγ mRNA expression, NIH/3T3 cells, 3T3-L1 preadipocytes and adipocytes (day 4) were treated with 5'-aza-cytidine (5 or 10 μM, Wako Pure Chemicals, Chuo-ku, Osaka, Japan) or trichostatin A (TSA, 400 nM, Wako Pure Chemicals) for 48 h, and total RNA was extracted for real time RT-PCR (see below). Demethylation of the PPARγ promoter was confirmed by *Hpy*CH4IV digestion following bisulfite conversion (described below) of genomic DNA prepared from the treated cells.

For adipose tissue preparation, 10 week-old C57BLKS/J *lar- +Lepr*^*db*^*/+Lepr*^*db *^and control C57BLKS/J *lar- m*^+^*/m*^+ ^mice were purchased from Japan SLC (Hamamatu, Shizioka, Japan), and 20 week-old C57BL/6J diet-induced obesity model mice, which had been fed a high-fat diet from 4 to 20 weeks of age, and control 20 week-old C57BL/6J mice fed a normal diet were purchased from Japan Charles River (Yokohama, Kanagawa, Japan). Inguinal subcutaneous adipose tissue and epididymal adipose tissues were extirpated from each mouse and snap frozen in liquid nitrogen. Three mice were killed for each experimental condition.

### Determination of DNA methylation status by bisulfite conversion

Genomic DNA from cultured cells and adipose tissues was prepared by standard phenol/chloroform extraction and ethanol precipitation. Bisulfite treatment of extracted genomic DNA was performed with an EZ DNA Methylation Gold Kit (Zymo Research, Orange, California, USA), following the manufacturer's instructions. The PPARγ promoter region in the bisulfite-converted genome was amplified by PCR using primers designed as follows: (region 1) forward, 5'-GATGTGTGATTAGGAGTTTTAATTAAAG-3'; reverse, 5'-CAAACCTAAATTAACTAACACTATCCTAAC-3', (region 2) forward, 5'-GTTAGGATAGTGTTAGTTAATTTAGGTTTG-3'; reverse, 5'-CATACAATTTCACCCACACATAAATAC-3', (site -437 bp) forward, 5'-GATGTGTGATTAGGAGTTTTAATTAAAG-3'; reverse, 5'-CCAAAACAAAAATTATTCAATATTAATTAC-3', (site -247 bp) forward, 5'-GAATAGTGAATGTGTGGGTTATTGG-3'; reverse, 5'-CAAACCTAAATTAACTAACACTATCCTAAC-3'. The promoter was divided into two regions (region 1 and 2) and amplified by the primer set named 'region 1' and 'region 2'. To investigate specific methylation status of CpG site -437 bp and -237 bp, two regions flanking to those sites was amplified by the primer set named 'site -437 bp' and 'site -237 bp'. DNA aliment sequencing was performed to examine the methylation status of the amplicon by primer set 'region 1' and 'region 2', and restriction enzyme digestion was performed to examine the amplicon of 'region 1', 'site -437 bp' and 'site -237 bp'.

For sequencing, PCR fragments were cloned into the pGEM T-Easy vector system (Promega, Madison, Wisconsin, USA), and eight clones were sequenced for each sample to determine methylation status. For restriction enzyme digestion analysis, PCR fragments corresponding to region 1, site -437 bp and site -247 bp were digested with *Hpy*CH4IV (New England Biolabs, Ipswich, Massachusetts, USA) and electrophoresed to separate the digested fragments (for region 1 fragments, at least one of -437 bp, -298 bp and -247 bp were methylated) and undigested fragments (for region 1 fragments, all of the three CpG sites were unmethylated). Because only unmethylated cytosine residues are converted to thymine by the bisulfite conversion reaction and following PCR, PCR fragments generated from unmethylated genomic DNA are resistant to *Hpy*CH4IV digestion, whereas those generated from methylated DNA are digested by the enzyme. The relative levels of the digested and undigested fragments were estimated following analysis of images of ethidium bromide stained agarose gels using ImageJ 1.38x software provided by the National Institutes of Health [32].

### Analysis of PPARγ expression by real time RT-PCR

Total RNA was extracted from cultured cells using the RNeasy Mini Kit (Qiagen, Hilden, Germany), or from adipose tissues using the RNeasy Lipid Tissue Mini Kit (Qiagen). Total RNA was used for first strand cDNA synthesis using a poly-T_20 _primer and the Transcriptor First Strand cDNA Synthesis Kit (Roche, Basel, Switzerland). The synthesized cDNA was used as a template for real time PCR using the LightCycler FastStart DNA MasterPLUS SYBR Green I (Roche). PPARγ mRNA (both PPARγ 1 and PPARγ2, or PPARγ2 exclusively) and β-actin mRNA were amplified using primers designed as follows: (PPARγ) forward, 5'-CGGTTTCAGAAGTGCCTTG-3'; reverse, 5'-GGTTCAGCTGGTCGATATCAC-3', (PPARγ2) forward, 5'-ATGCACTGCCTATGAGCACT-3'; reverse, 5'-CAACTGTGGTAAAGGGCTTG-3', (β-actin) forward, 5'-AGCTATGAGCTGCCTGACGG-3'; reverse, 5'-CCAGACAGCACTGTGTTGG-3'. Template amounts of each PPARγ/β-actin mRNA were calculated by kinetic analysis of PCR amplification. The level of expression of PPARγ mRNA was normalized to the level of β-actin mRNA expression for comparisons between each sample.

### Chromatin immunoprecipitation assays

Chromatin immunoprecipitation assays were performed using the Chromatin Immunoprecipitation Assay Kit (Millipore, Billerica, Massachusetts, USA) according to the manufacturer's instructions. In brief, 10^6 ^cells were treated with 1% formaldehyde for 10 min at 37°C to form DNA-protein crosslinks. Each sample was then sonicated on ice and incubated with antibody at 4°C overnight. PCR was performed for 30 cycles on DNA extracted from the immunoprecipitated DNA-protein complexes. The amount of each PCR product was evaluated by ethidium bromide staining following electrophoresis through an agarose gel. The following primers were used to amplify the PPARγ promoter: forward, 5'-CCAAATACGTTTATCTGGTGTTTC-3'; reverse, 5'-CGTTGCTACATTGTCTCGC-3'. Antibodies used were as follows: MeCP2 (Abcam, Cambridge, Massachusetts, USA), MBD1 (Epigentek Group, Brooklyn, New York, USA), MBD2a (Novus Biologicals, Littleton, Colorado, USA), dimethylated Histon H3K9 (Abcam), dimethylated Histone H3K4 (Abcam).

### Luciferase reporter assays

The 5'-flanking region of the PPARγ gene (-987 to +17) was amplified by PCR from genomic DNA prepared from 3T3-L1 cells, and the fragment was cloned into the pGL4.12 vector encoding a firefly luciferase reporter gene (Promega). Amplification of the reporter construct was carried out using the *dam-/dcm- Escherichia coli *strain (New England Biolabs). To obtain a methylated reporter, the construct was incubated with 3 units/μg SssI methylase (New England Biolabs) in the presence of 160 μM S-adenosylmethionine at 37°C for 3 h. Completion of the methylation was confirmed by resistance to *Hpy*CH4IV digestion.

NIH/3T3 cells, 3T3-L1 preadipocytes and adipocytes (day 4) were transfected or electroporated with the firefly luciferase reporter construct using the FuGENE 6 reagent (Roche), the Cell Line Nucleofector Kit V (Amaxa, Walkersville, Maryland, USA) and the Cell Line Nucleofector Kit L (Amaxa), respectively. To normalize the luciferase activity, an amount of control plasmid pGL4.74 (Promega) encoding a *Renilla reniformis *luciferase gene, corresponding to 10% of the amount of the text plasmid, was cotransfected into the cells. The activities of both luciferases were determined using a Dual Luciferase Reporter System (Promega) according to the manufacturer's instructions.

## Authors' contributions

KF conceived and designed the study, carried out all the collection, analysis and interpretation of data, and drafted the manuscript. FK contributed to the design of the study and the technical direction of the experiments. KS contributed to the design of the study and the revising of the manuscript. MM contributed to the conception and design of the study, and the preparation of the manuscript. All authors read and approved the final manuscript.

## Supplementary Material

Additional file 1 **Trichostatin A treatment of NIH/3T3 cells**. The expression profile of peroxisome proliferators activated receptor γ (PPARγ) mRNA in trichostatin A (TSA)-treated NIH/3T3 cells. The cells were exposed to TSA at the indicated concentrations for 48 h in growth medium. The expression levels of PPARγ mRNA and β-actin mRNA were determined by real time reverse transcriptase polymerase chain reaction (RT-PCR). The level of PPARγ mRNA was normalized to that of β-actin, and the relative normalized levels are shown at the left part. Results of real time RT-PCR experiments of 5'-aza-cytideine (5'-aza-C) treatment of cells (these results are also presented in Figure 2a) are also shown at the right part, as a comparison. Data represent the mean ± SD of three independent experiments performed in triplicate. Click here for file

Additional file 2 **Chromatin immunoprecipitation assays of peroxisome proliferators activated receptor γ (PPARγ) promoter region in NIH/3T3**. A total of 10^6 ^NIH/3T3 cells were harvested and used as the input for each assay. DNA fragments immunoprecipitated by the indicated antibody were recovered and amplified by the primers designed for the PPARγ promoter region (see Figure 1). A total of 1% of the input was also amplified without chromatin immunoprecipitation (ChIP) and is shown at the left. Click here for file

Additional file 3 **The weight of body and extirpated tissues of the four kinds of mouse**. Three mice were killed for each type, and one of the three was measured. For subcutaneous adipose tissue (SAT), only the inguinal part was extirpated and tested. Click here for file

Additional file 4 **A comparison of the sequence alignment of human and murine peroxisome proliferators activated receptor γ (PPARγ) promoter**. The alignment of 1,000 bp upstream of the start codon of PPARγ2 is compared using CLUSTALW (v. 1.83, provided by DNA Data Bank of Japan: . The methylation sites are highlighted in each species, and their position relative to the transcription start site (TSS) (see main text) is indicated in the murine genome. Legends are listed below. GenBank accession codes: NC_000003, NC_000072. Click here for file
