# Supplementary figures and images for: Expression of the peroxisome proliferator activated receptor γ gene is repressed by DNA methylation in visceral adipose tissue of mouse models of diabetes
Source: BMC Biol. 2009 Jul 10;7:38. doi: 10.1186/1741-7007-7-38 (PMC2715379; doi:10.1186/1741-7007-7-38)

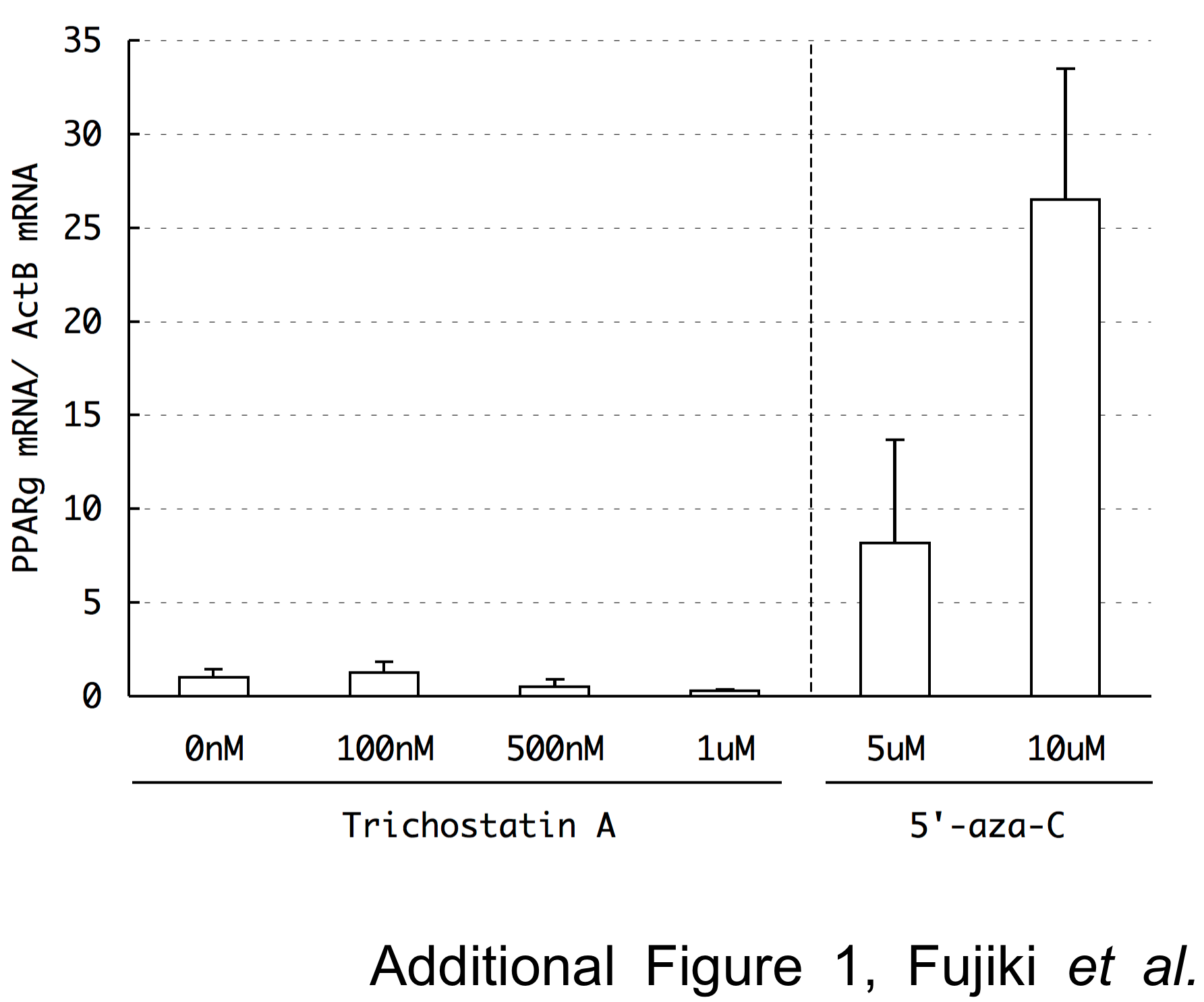

Supplement: Additional file 1 — Trichostatin A treatment of NIH/3T3 cells. The expression profile of peroxisome proliferators activated receptor γ (PPARγ) mRNA in trichostatin A (TSA)-treated NIH/3T3 cells. The cells were exposed to TSA at the indicated concentrations for 48 h in growth medium. The expression levels of PPARγ mRNA and β-actin mRNA were determined by real time reverse transcriptase polymerase chain reaction (RT-PCR). The level of PPARγ mRNA was normalized to that of β-actin, and the relative normalized levels are shown at the left part. Results of real time RT-PCR experiments of 5'-aza-cytideine (5'-aza-C) treatment of cells (these results are also presented in Figure 2a) are also shown at the right part, as a comparison. Data represent the mean ± SD of three independent experiments performed in triplicate. [file 1741-7007-7-38-S1.tiff]

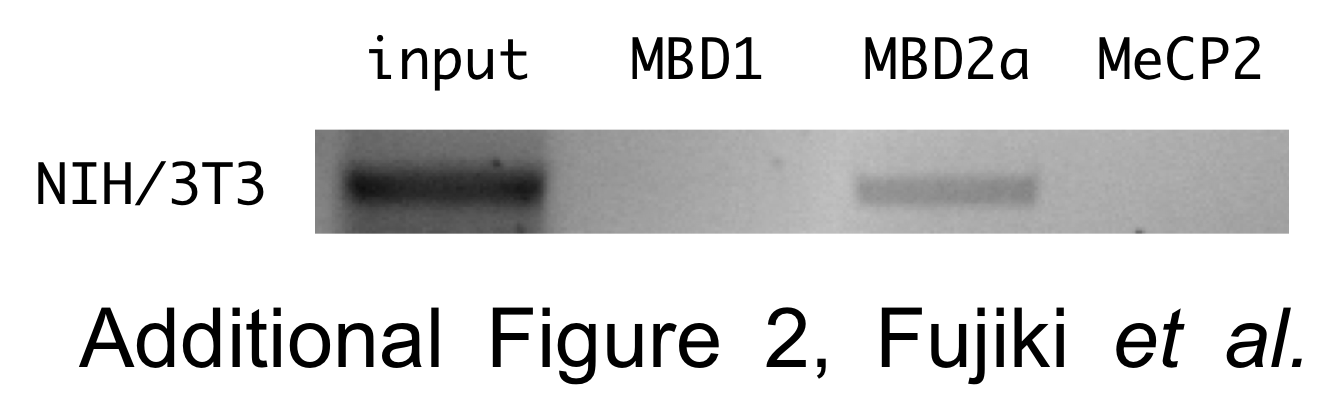

Supplement: Additional file 2 — Chromatin immunoprecipitation assays of peroxisome proliferators activated receptor γ (PPARγ) promoter region in NIH/3T3. A total of 106 NIH/3T3 cells were harvested and used as the input for each assay. DNA fragments immunoprecipitated by the indicated antibody were recovered and amplified by the primers designed for the PPARγ promoter region (see Figure 1). A total of 1% of the input was also amplified without chromatin immunoprecipitation (ChIP) and is shown at the left. [file 1741-7007-7-38-S2.tiff]

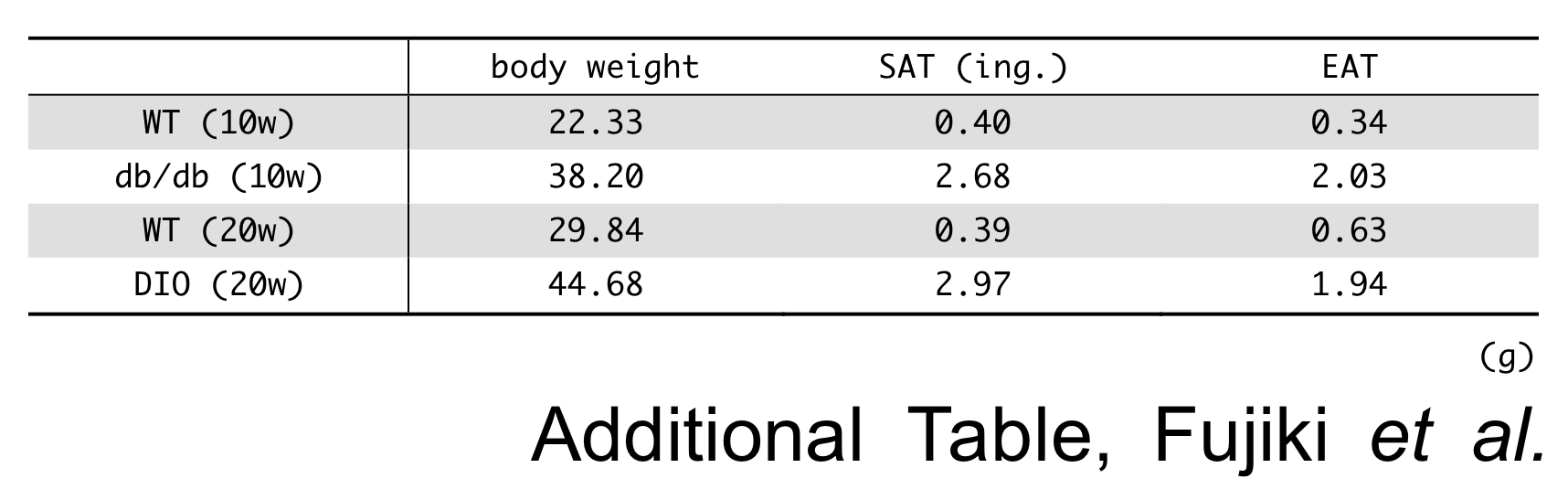

Supplement: Additional file 3 — The weight of body and extirpated tissues of the four kinds of mouse. Three mice were killed for each type, and one of the three was measured. For subcutaneous adipose tissue (SAT), only the inguinal part was extirpated and tested. [file 1741-7007-7-38-S3.tiff]

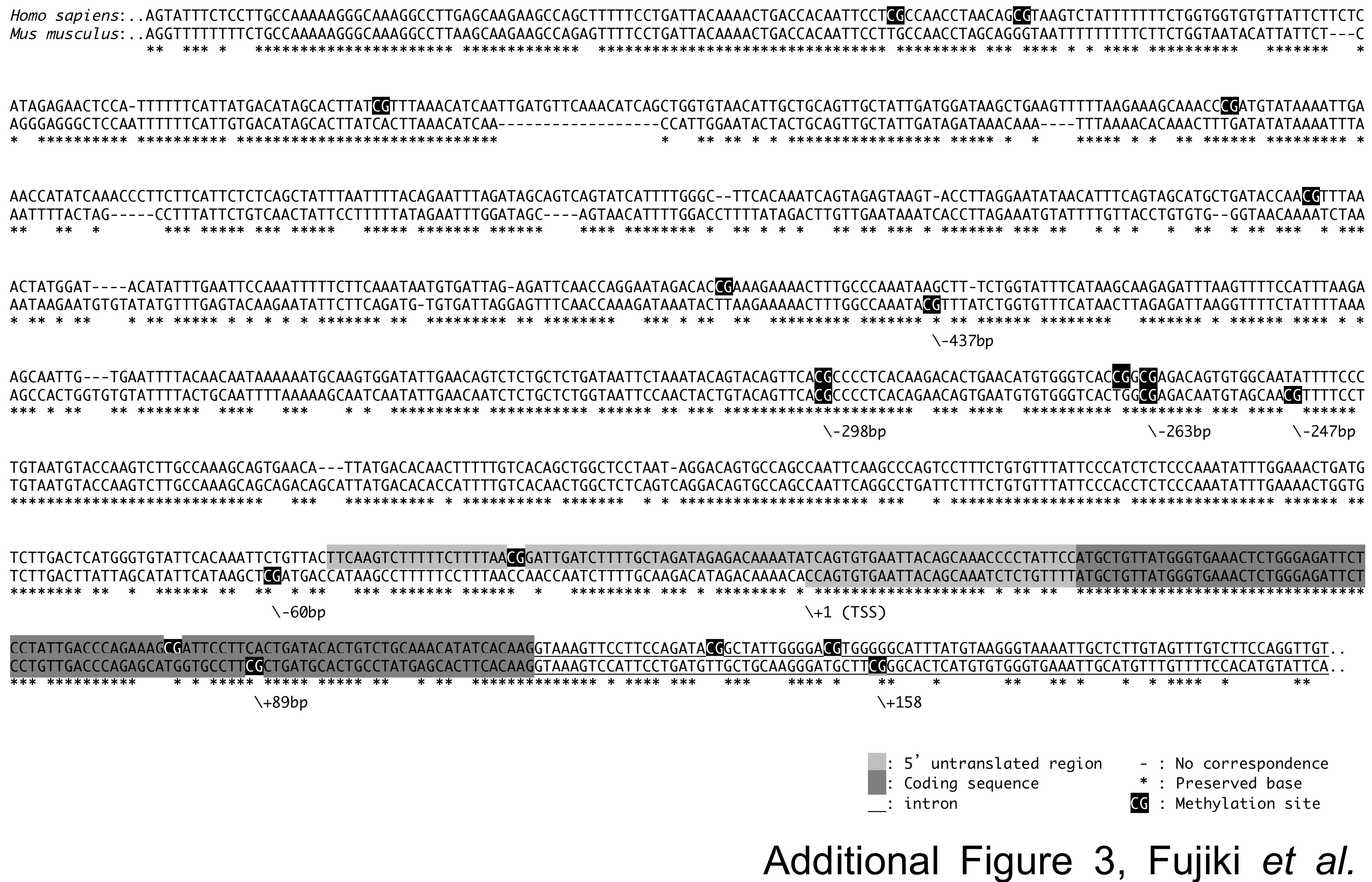

Supplement: Additional file 4 — A comparison of the sequence alignment of human and murine peroxisome proliferators activated receptor γ (PPARγ) promoter. The alignment of 1,000 bp upstream of the start codon of PPARγ2 is compared using CLUSTALW (v. 1.83, provided by DNA Data Bank of Japan: . The methylation sites are highlighted in each species, and their position relative to the transcription start site (TSS) (see main text) is indicated in the murine genome. Legends are listed below. GenBank accession codes: NC_000003, NC_000072. [file 1741-7007-7-38-S4.tiff]
